# Timing of Exposure and Bisphenol-A: Implications for Diabetes Development

**DOI:** 10.3389/fendo.2018.00648

**Published:** 2018-10-31

**Authors:** Eva Tudurí, Laura Marroqui, Reinaldo S. Dos Santos, Iván Quesada, Esther Fuentes, Paloma Alonso-Magdalena

**Affiliations:** Centro de Investigación Biomédica en Red de Diabetes y Enfermedades Metabólicas Asociadas and Instituto de Biología Molecular y Celular, Universidad Miguel Hernández de Elche, Alicante, Spain

**Keywords:** Bisphenol-A, diabetes, pancreatic β-cell, timing of exposure, metabolic programming

## Abstract

Bisphenol-A (BPA) is one of the most widespread endocrine disrupting chemicals (EDCs). It is used as the base compound in the production of polycarbonate and other plastics present in many consumer products. It is also used as a building block in epoxy can coating and the thermal paper of cash register receipts. Humans are consistently exposed to BPA and, in consequence, this compound has been detected in the majority of individuals examined. Over the last decade, an enlarging body of evidence has provided a strong support for the role of BPA in the etiology of diabetes and other metabolic disorders. Timing of exposure to EDCs results crucial since it has important implications on the resulting adverse effects. It is now well established that the developing organisms are particularly sensitive to environmental influences. Exposure to EDCs during early life may result in permanent adverse consequences, which increases the risk of developing chronic diseases like diabetes in adult life. In addition to that, developmental abnormalities can be transmitted from one generation to the next, thus affecting future generations. More recently, it has been proposed that gestational environment may also program long-term susceptibility to metabolic disorders in the mother. In the present review, we will comment and discuss the contributing role of BPA in the etiology of diabetes. We will address the metabolic consequences of BPA exposure at different stages of life and comment on the final phenotype observed in different whole-animal models of study.

Diabetes mellitus is among the most prevalent metabolic disorders and the current number of cases is approaching epidemic proportions. According to the latest Global Burden Disease report (2015), the prevalence of diabetes has risen from 333 million people in 2005 to approximately 435 million in 2015 (1). The predictions suggest that it will be the seventh leading cause of death in 2030 (2). As proposed by the American Diabetes Association (ADA), the classification of this pathology includes type 1 (T1DM), type 2 (T2DM) and gestational diabetes mellitus (GDM), as well as other forms of diabetes (3). Despite differences on the underlying causing factors, all of them lead to hyperglycemia, which results from defects on insulin secretion, insulin resistance or a combination of both (4).

More than 90% of patients with diabetes have T2DM (5). The etiology of T2DM is multifactorial with a clear genetic background but also with an important environmental compound (6). Poor nutrition, lack of exercise and overweight are commonly seen as contributing factors to cause T2DM but other environmental factors like endocrine disrupting chemicals (EDCs) are also key. EDCs are defined as any chemical or mixture of chemicals able to disrupt any aspect of endocrine function (7). They constitute a heterogeneous range of compounds widely used in consumer products including food packaging, personal care products, pesticides or building materials, among others. General population is exposed to EDCs through different routes, mainly the oral, dermal or inhalation ones (8). From a mechanistic point of view, it is well described that they can interfere with hormonal signaling pathways principally by binding to hormone receptors and/or altering hormone production, metabolism and transport (8, 9). Over the last decade, burgeoning scientific evidence have disclosed that a subset of EDCs are able to modify sensitive metabolic processes leading to altered energy homeostasis and/or glucose and lipid metabolism (10–12). One of the best documented example is the case of bisphenol-A (BPA), firstly synthetized by Dianin in 1891 and reported to show estrogenic properties in 1930s (13). BPA is a monomer that is used as the base compound in the manufacture of polycarbonate and other plastics, and it is also employed as a building block in epoxy can coating and the thermal paper of cash register receipts. It became one of the most pervasive chemical in modern life from the moment it was introduced in the plastic industry in the 50s. Today, it constitutes one of the highest volume chemical produced widespread, with over 6 billion pounds manufactured every year. BPA has been found to migrate from polycarbonate containers and leach into food or water because of overheat, the presence of acidic or basic conditions and/or a repetitive use. Thermal paper has also been shown to constitute an important source of exposure to this EDC (14). The ubiquitous nature of BPA makes human exposure to occur on a virtually constant basis. In consequence, it has been detected in the majority of individuals examined (15, 16).

As regards the regulatory aspects, back in the 80s the Environmental Protection Agency (EPA) established the lowest adverse effect level (LOAEL) for BPA at 50 mg/kg/day and, based on that, the safety level was set on 50 μg/kg/day (17). Although many studies have shown deleterious effects of BPA at doses below the calculated safe dose (8, 18), the reference value of 50 μg/kg/day is still in force. For the European Food Safety Authority the tolerable daily intake has been recently established in 4 μg/kg/day (19). Remarkably, both the reference dose and the tolerable daily intake are higher than the 95th percentile BPA intake level for adults (1.5 μg/kg/day) estimated by the World Health Organization (WHO). This estimation has been done based on published exposure estimation in several countries and regions, as well as taking into account the information available regarding information on food consumption patterns, and BPA presence in foods relevant to the population groups (20).

## Overview of the molecular mechanisms linking BPA exposure and diabetes

Current molecular and cellular evidence support that BPA may augment diabetes risk by affecting pancreatic β-cell function and/or insulin action in different metabolically active tissues (8, 10, 21). Before commenting on that, we will briefly described some general aspects of BPA mode of action.

BPA is mainly considered a xenoestrogen due to its capacity to promote estrogen-like activities. Structurally, BPA has two phenolic and two (4, 40)-OH substituents that confer the ability to join into the ER-binding structure. Thus, BPA can bind to both estrogen receptor α (ERα) and β (ERβ) although with 1,000 to 2,000-fold less affinity than the natural hormone 17-β-estradiol (E2) (22, 23). Based on that, BPA was considered a weak estrogen for many years. Nevertheless, today we know that, when acting through non-classical ER pathways, BPA can elicit responses with the same potency than E2. Non-classical ER pathways mainly include nuclear mechanisms of estrogen responses elicited by recruiting different transcriptional co-activators or co-repressors, as well as extranuclear-initiated signaling pathways which regulate different cellular pathways such as, gene transcription as well as ion channel and kinase activity. We here summarize some of the principal mechanisms behind BPA action triggered via non-classical estrogen pathways. For more information readers are referred to the following reviews (24–28).

Scientific evidence support that BPA-dependent estrogenic activity flows, at least in part, through rapid, non-genomic alterations of Ca^2+^ handling. Both BPA and E2, in the pico and nanomolar range, have shown to rapidly increase [Ca^2+^] in GH3/B6 pituitary tumor cells resulting in prolactin secretion (29). In a similar manner, BPA, at exactly the same doses than E2 (100 pM-1nM), potentiated Ca^2+^ signaling and insulin release in pancreatic β-cells (30, 31), while in pancreatic α-cells BPA mimicked E2 action by inhibiting low-glucose induced [Ca^2+^] oscillations (32). Importantly, *in vivo* studies also reported rapid insulin release in response to low doses of BPA (33). Rapid effects of BPA have also been observed in cardio myocytes. Low dose concentrations of BPA and E2 induced arrhythmic actions in isolated ventricular myocytes from female rats by altering Ca^2+^ handling (34); an effect that depends on the balance between ERα and ERβ (35). These BPA actions on cardio myocytes were characterized by non-monotonic dose responses (36).

Another major mechanism of BPA-induced extranuclear signaling pathways involves the activation of ERK/MAPK kinases. A wide range of BPA concentrations have been shown to rapidly increase the phosphorylation state of ERK1/2 in developing cerebellar neurons (37). In pancreatic islets of Langerhans BPA increased insulin content with the same potency than E2 acting through ERα. This was not an acute but a long-term effect which required the activation of ERK (38).

Furthermore, BPA showed binding affinities for GPR30, a relatively novel 7-transmembrane estrogen receptor (39). Several data support BPA action within nanomolar range via GPR30 in several systems. For example, BPA led to testicular seminoma cell proliferation through GPR30 (40). In pancreatic cells, BPA promoted insulin secretion dysfunction which was connected with inhibition of Pdx-1 and decreased expression of miR-338 (41).

Overall, it has been proposed that these plethora of mechanisms could account for the low dose effects of BPA that have been defined as “any biological change occurring in the range of typical human exposures or at doses lower than tested in traditional toxicological assessments” (42). Hundreds of studies have been published over the years providing strong evidence for low dose BPA effects having the potential to pose health hazards. These low dose effects have been observed for a variety of endpoints including metabolic parameters but also prostate weight, spermatogenesis, brain and mammary gland development as well as hormone levels, among others (18, 43). In addition, it is important to highlight that BPA, like hormones, do not obey the Paracelsus principle of “the dose makes the poison.” On the contrary, a typical feature of BPA actions includes non-monotonic dose response curves (NMDRCs) often manifested as U- or inverted U-shaped curves. The mechanisms behind these NMDRCs are typically related to cytotoxicity, receptor selectivity, receptor down regulation or competition with endogenous hormones (44). In addition to its estrogenic activity, BPA may act as an antagonist of androgenic (at doses in the range of 100 nM-10 μM) (45) and thyroid (1–100 μM) (46) activity, and may also activate the human estrogen-related receptor (26 nM) (47), pregnane receptor (2 μM) (48) and the glucocorticoid receptor (1 μM) (49).

A large number of cellular studies have demonstrated that BPA targets pancreatic β-cells. Environmental relevant doses of BPA have been shown to modulate K_ATP_ channel activity, which is key in the stimulus-secretion coupling process that culminates with insulin secretion. In both mouse and human islets, BPA provoked a rapid closure of the K_ATP_ channel and subsequently, increased insulin release. This effect was mediated by the extranuclear activation of the estrogen receptor beta (ERβ) (30, 50). Additionally, BPA may enhance glucose-induced insulin biosynthesis and content via binding to extranuclear ERα (38). In several models of β-cell lines, BPA has also been reported to affect insulin release. In the nanomolar range, it enhances basal (51, 52) and glucose-stimulated insulin secretion (GSIS), leading to ER stress. Conversely, at higher doses in the micromolar range, it displays the opposite effect (53). Furthermore, BPA treatment has been shown to increase reactive oxygen species production and DNA damage (54) as well as to augment apoptosis (53).

BPA may also alter glucose regulation by disrupting insulin action in different peripheral metabolic tissues. In the murine adipose cell line 3T3-L1 and in human adipocytes, BPA impaired insulin-stimulated receptor phosphorylation and signaling, which led to decreased insulin sensitivity and glucose disposal (55, 56). BPA treatment also provoked an up-regulation of inflammatory signaling pathways (56) and induced adipogenesis (57–59).

BPA has also been reported to exert direct effects on hepatocyte physiology. In different hepatic cell lines, BPA has been shown to induce lipid accumulation, favoring the development of hepatic steatosis (58, 60).

## Relevance of exposure timing: the concept of metabolic programming

David Barker and colleagues suggested for the first time that early-life nutrition is associated with a number of offspring health outcomes including coronary heart disease, hypertension and T2DM (61–63). These first observations established the bases of the fetal origins of adult disease hypothesis, which states that events occurring during early development may increase the risk of specific diseases later in life. This concept was later expanded to other aspects of developmental plasticity such as early postnatal period and intergenerational influences, and set the framework of the Developmental Origins of Health and Disease (DOHaD) theory (64).

Overall, this paradigm relies on the fact that there exist specific developmental periods whereby an organism is more sensitive to environmental insults. While those insults were initially referred to nutritional imbalance, today it is well demonstrated that the exposure to some environmental chemicals during development may also lead to increased incidence to non-communicable diseases like T2DM (65). The understanding of the molecular mechanisms behind this phenomenon is far to be complete. Nevertheless, it has been proposed that environmental agents acting during specific windows of sensitivity may promote changes in gene expression, cell metabolism, hormone regulation or cellular plasticity, among others. Such functional changes might permanently modify the organism's physiologic and metabolic homeostatic set points, programming the individual to later risk for metabolic diseases (11).

So far, epigenetics processes have been proposed to be the major driving mechanism in the altered programming during development. These epigenetic changes entail chemical modifications of DNA which are heritable and reversible but with no changes in the underlying DNA sequence. Rather, epigenetic marks are capable to alter DNA accessibility and chromatin structure and, therefore, regulate gene expression patterns. They comprise DNA methylation, histone modification, states of chromatin condensation or non-coding RNA-associated gene silencing (66–69). As it will be described later, early life exposure to BPA has been shown to induce epigenetic alterations associated with diabetogenic outcomes.

## Metabolic consequences of developmental exposure to BPA

A review of the currently available literature reveals increasing number of animal studies exploring BPA effects on metabolic developmental programming. Although most of the studies differ in the route and timing of BPA exposure (gestation or gestation and lactation), dose, and animal strain used, they generally strengthen the evidence of the diabetogenic action of BPA. Here we review studies reported in the literature addressing the contributing role of BPA in the etiology of diabetes. Detailed information on dosage, age, gender and timing of exposure can be found in Supplemental Table 1.

Initial observations came in 2010 when it was shown that the treatment of pregnant dams on days 9–16 of gestation with two different doses of BPA (10 or 100 μg/kg/day) resulted in impaired glucose homeostasis in male adult offspring. Of note, the metabolic phenotype observed was different depending on the BPA dose administered. Thus, animals intrauterine exposed to the lowest dose showed marked insulin resistance, hyperinsulinemia, altered glucose tolerance and increased triglyceride levels. Pancreatic β-cell function was also found to be affected as reflected by enhanced GSIS, both *in vivo* and *in vitro*, probably as a compensatory mechanism for the insulin resistance developed. On the contrary, animals exposed to the highest dose showed mild glucose intolerance with no changes on insulin sensitivity (70).

Later on, a wider BPA dose range study came out with doses tested from 10-fold below EPA reference dose (50 μg/kg/day) to 10-fold above the no-observed-adverse-effect level (NOAEL, 5,000 μg/kg/day). Offspring's metabolic changes following oral exposure of pregnant dams to BPA (5, 50, 500, 5,000, and 50,000 μg/kg/day, days 9–18 of gestation) were evaluated in CD1 mice. The authors found a non-monotonic dose response for body weight changes and increased adipocyte number and volume in the offspring. In addition, offspring from all BPA groups, except for the highest dose one, developed glucose intolerance. Intrauterine BPA-exposed animals to 5 μg/kg/day displayed higher plasma insulin levels, and those treated with the doses of 5 and 5,000 μg/kg/day showed insulin resistance (71).

A similar study by Liu and collaborators focused on a range of timing of exposure instead of on a range of doses. Preimplantation (days 1–6 of pregnancy), fetal (P6-PND0), neonatal (PND0-PND21) or fetal and neonatal (P6-PND21) periods were explored with the same dose of BPA (100 μg/kg/day by subcutaneous injection). The authors reported changes on body weight that were dependent on the animal gender and the exposure timing to BPA. Regarding glucose metabolism, female mice exposed to BPA during fetal and preimplantation periods manifested glucose intolerance at the age of 3 and 6 months. In the case of male offspring, glucose intolerance was observed in fetal, neonatal, and fetal and neonatal exposed-groups at 3 months of age and persisted at 6 and 8 months of age in the first group. Insulin sensitivity was also altered. Reduced insulinogenic index was observed in the 3 month-old female fetally BPA-exposed as well as in males receiving BPA in the fetal, neonatal and fetal and neonatal periods. Together with changes in the insulinogenic index, these animals showed attenuated response to insulin that only persisted in the male fetally exposed-group up to 8 months of age. A transient impairment of pancreatic β-cell function was observed in *in vitro* assays, which was thought to be a functional issue rather than morphological abnormalities, since pancreatic β-cell mass remained invariable or even increased. Isolated islets released less insulin in response to glucose in the fetal female as well as in the fetal, neonatal, and fetal and neonatal male exposed groups. Nevertheless, no changes on insulin secretion were observed when animals reached 8 months of age (72). Of note, in a similar experimental animal model, BPA promoted impaired insulin signaling and glucose transport expression in the prefrontal cortex at 8 months of age (73). Furthermore, glucose intolerance in adult BPA offspring has also been connected with alterations in the structure of hypothalamic energy balance circuitry (74, 75).

In another study, pregnant rats were exposed to 1 and 10 μg/mL BPA (drinking water) from gestation day 6 through lactation, and then male offspring was studied at postnatal days 50 (PND50) and 100 (PND100). Increased body weight was manifested on PND7 and persisted at PND100 together with hyperglycemia, insulin resistance, decreased adiponectin levels and increased oxidative stress (76).

Not only murine but also large-animal model-based studies have found postnatal metabolic outcomes related to fetal exposure to BPA at a level relevant to human exposure. In line with this, Veiga-Lopez and collaborators explored pregnancy as a critical period for BPA exposure by treating female sheep with 50, 500, or 5,000 μg/kg/day (subcutaneous injection) from days 30–90 of gestation. Increased fasting blood glucose in the lowest BPA dose group and a marked tendency to decreased insulin sensitivity in all BPA groups were found in the female offspring at 6 weeks of age. In the postpubertal period (13 months of age), insulin resistance was observed in the 500 μg/kg/day BPA-exposed group. Moreover, enhanced mean adipocyte area and diameter in the visceral adipose tissue, as well as increased inflammation in the subcutaneous adipose tissue depot were reported (77).

It should be mentioned that some reports did not find developmental BPA effects on glucose regulation. This is the case of Ryan and collaborators' article, in which perinatal pregnant CD-1 mice were exposed to BPA (1 ppb via the diet) from embryonic day 0 until weaning. The authors observed that BPA mice were heavier and longer at the moment of weaning but the body weight returned to normal levels in adulthood compared to controls. They did not find any effects on glucose tolerance neither at 8 nor 15 weeks of age. No other metabolic parameters such as insulin sensitivity or GSIS were analyzed. It is remarkable that the authors did not explore more advanced ages in which metabolic abnormalities were revealed in other animal studies (78). In a similar manner, no impairment on glucose tolerance was observed in male 20 week-old offspring after BPA perinatal exposure, although dose-dependent increases of body weight were reported with no changes on global hepatic DNA methylation (79, 80).

Intrauterine exposure to low BPA doses has been shown to affect not only pancreatic β-cell function in the adult offspring but also pancreas development. Increased pancreatic β-cell mass has been found in BPA-exposed animals at the moment of birth, which was also observed at the moment of weaning and PND30. This early life alteration was mainly related to overexpression of cell cycle genes, culminating with a rise in β-cell division as well as decreased apoptosis. All together, these abnormalities led to marked hyperinsulinemia. It is noteworthy to mention that this excessive insulin signaling has been proposed to be crucial for the metabolic phenotype observed in BPA animals later in life (81). Furthermore, BPA has been shown to have an impact on pancreas morphology with a greater number of islet-cell clusters at embryonic day 18.5 (82).

In order to reveal and/or exacerbate any underlying metabolic disorder that accounts for BPA action, the so-called high fat diets (HFD) have been used in several laboratory studies. This approach has helped to shed some light into the metabolic derangements following prenatal BPA exposure. In one study, pregnant rats were exposed to BPA at doses of 50, 250, or 1,250 μg/kg/day by oral gavage through gestation and lactation, and later, offspring was fed with either standard diet (STD) or HFD from the moment of weaning. While some detrimental effects were observed at 26 weeks of age on the BPA animals fed with STD, those effects were accelerated and exacerbated by HFD and became apparent at the age of 15 weeks. Increments in body weight, insulin plasma levels, GSIS and pancreatic β-cell mass were observed in the BPA lowest dose fed with STD group. The same group of animals challenged with HFD exhibited glucose intolerance, hyperglycemia, dyslipidemia and hyperleptinemia. Remarkably, no adverse effects were observed at the highest doses (83). In a different report, pregnant dams were subcutaneously treated with BPA (10 μg/kg/day) from day 9 to 16 of gestation and then 1-month-old male offspring were maintained during 13 or 24 weeks on STD or HFD. In BPA-treated mice, body weight at birth was decreased followed by catch-up growth with similar weight than HFD mice at 22 weeks of age. At the 17th week, BPA animals showed fasting hyperglycemia, increased NEFA and insulin levels, and a tendency to be glucose intolerant, which was severely impaired at the 28th week in STD. In addition, intrauterine exposure to BPA led to changes in the expression of genes involved in glucose and lipid metabolism in liver and adipose tissue as well as in pancreatic β-cell function that were similar to those observed with HFD treatment (84). Similar metabolic outcomes have also been observed in sheep, including insulin resistance, increased adipocyte size and deposition, and inflammation in BPA-exposed and postnatal overfeeding groups (77). Furthermore, metabolomic analysis revealed changes in metabolic profiling related to prenatal BPA exposure, such as variations in glucose, pyruvate, some amino acids and neurotransmitters, which were evident soon after birth (85, 86).

As stated before, epigenetics has been outlined as one of the major links between genes, environment, and developmental outcomes. Several articles have shown that gestational exposure to environmentally relevant BPA doses are able to promote epigenetic changes in the progeny that correlate to adverse multigenerational health effects. Thus, at the age of 3 weeks, male offspring perinatally exposed to BPA (50 μg/kg/day) exhibited overexpression of the methyltransferase 3B mRNA. This phenomenon was related to decreased hepatic global DNA methylation and resulted in reduced hepatic glucokinase gene expression. No changes in insulin sensitivity were found at this age. However, at 21 weeks of age, impaired glucose tolerance and insulin resistance showed up, suggesting that the global methylation changes contributed to the metabolic disarrangement observed in adulthood (87). Epigenetic changes have been connected not only with F1 generation but with subsequent generational effects. In line with this, decreased insulin sensitivity and glucose intolerance were found in F2 generation. These effects were proposed to be transmitted through the male germ line and to be associated with changes in glucokinase DNA methylation (88). Later on, more evidence came out confirming multigenerational phenotypic inheritance upon perinatal BPA exposure. Pregnant mice were treated with two different doses of BPA (10 μg/kg/d and 10 mg/kg/day) and the effects on the offspring were studied. Overall, increased body weight, glucose intolerance and reduced GSIS were observed in both F1 and F2 male offspring. Importantly, it was demonstrated for the first time that maternal glucose homeostasis was affected in F0 but not in F1 pregnant mice, suggesting that the alterations observed were not caused by an abnormal maternal metabolic milieu but were rather related to epigenetic changes. In particular, the authors reported fetal increased expression of the imprinted Igf2 gene and enhanced DNA methylation at the Igf2 differentially methylated region 1 both in F1 and F2 embryos (89). In the same year, another report confirmed the presence of glucose intolerance and pancreatic β-cell dysfunction in male BPA F2 generation; abnormalities that were associated with the dysregulation of Igf2/H19 in the islets of Langerhans (90). More recently, relying on the same epigenetic bases, metabolic effects found in F1 and F2 generation have been extended to impaired mitochondrial function and insulin secretion, reduced β-cell mass and islet inflammation (91).

These above-mentioned multigenerational effects differ from the transgenerational ones. While the former has been defined as “an exposure that directly influences multiple generations”, the latter implies transmission across generations but with no direct exposure or involvement, and are manifested from F3 generation. Up to date, much of the work has focused on outcomes in the BPA-exposed F1 generation or the intergenerational F2, but little is known about the transgenerational (F3 and beyond) outcomes, with just some evidence of embryonic exposure to BPA and obesogenic effects in F3 (92, 93).

Contrary to the case of T2DM, the experimental data supporting the contributing role of BPA in the developmental programming of T1DM is still very scarce. One study published in 2014 addressed this issue by using non-obese diabetic (NOD) mice, an animal model of T1DM. The study described how transmaternal orally-exposed female offspring (10 mg/L drinking water) showed aggravated severity of insulitis together with increased apoptosis and decreased number of resident macrophages at 11 weeks of age. This determined increased prevalence of diabetes later at 20 weeks of age (94).

Regarding the possible interactions with the immune system, perinatal exposure to BPA (50 μg/kg/day) has also been shown to promote systemic immune imbalances in male adult mice offspring. Remarkably, these alterations occurred in parallel to microbial disturbances and impaired glucose tolerance. The authors proposed that these disturbances precede the development of obese phenotype as the animals get old (95).

## Metabolic consequences of adult exposure to BPA

Multiple studies conducted in adult animal models have observed alterations in glucose and lipid metabolism following BPA treatment. Firstly, one of the most described outcomes is the insulinotropic action of BPA, i.e. its ability to stimulate insulin release from pancreatic β-cells. A single injection of BPA at a low dose of 10 μg/kg of body weight potentiated plasma insulin levels in mice within just 30 min (33). Likewise, BPA treatments with different durations and a wide range of doses, from 5 μg/kg/day to 20 mg/kg/day, also increased plasma insulin levels (33, 41, 96–99). Accordingly, further studies in islets from BPA-treated mice showed an increase in several parameters including GSIS (96), enhanced insulin content (33, 41), improved β-cell area and mass (41), and augmented islet Pdx1 transcript and protein levels, and NeuroD mRNA levels (41). It is important to note that hyperinsulinemia may eventually lead to insulin resistance and, therefore, may contribute to obesity and T2DM development (33, 100). In contrast, other reports described decreased plasma insulin levels after exposing mice to 100 μg/kg/day BPA for 20 days (101) or 28 days (102), or even unchanged plasma insulin levels after 8 months of BPA administration (5–5,000 μg/kg/day) (103). These discrepancies in the observed outcomes might be due to the use of different mouse strains and/or variations in the administration protocol. Secondly, BPA-treated adult mice displayed impaired glucose tolerance after 4 days receiving BPA at 100 μg/kg/day (33) and after 2 months of BPA treatment with 500 μg/kg/day (41) and 5,000 μg/kg/day (103). One study reported no changes in the glucose excursions in response to a glucose load after 8 days of treatment with BPA 100 μg/kg/day (96). Furthermore, some reports also described insulin resistance following BPA exposure (33, 41, 96). Overall, these effects on glucose homeostasis suggested that, besides the endocrine pancreas, other tissues involved in the regulation of glucose metabolism also displayed alterations. In fact, the insulin signaling pathway was altered in skeletal muscle and liver from BPA-treated mice, which showed diminished insulin-stimulated phosphorylation of the insulin receptor β subunit and Akt phosphorylation (96, 97, 104). Such failure in hepatic insulin signaling seems to be responsible for the impaired hepatic glucose oxidation and glycogen content found in rats treated with high doses of BPA (20 and 200 mg/kg/day) for 30 days (97). Furthermore, hepatic glucokinase activity was acutely (2 h) suppressed in response to an oral BPA bolus of 50 μg/kg (105). Additional analysis with liver samples showed that long-term exposure to BPA (20–200 mg/kg/day) potentiated the mRNA and protein levels of GLUT2 (97), as well as important factors involved in lipogenesis and biosynthesis of cholesterol when employing doses in the range of 5–5,000 μg/kg/day BPA (99, 103). Accordingly, augmented plasma triglycerides and cholesterol have been detected following BPA administration (0.5 and 2 mg/kg/day) during 4 weeks (103, 106), and hypercholesteronemia remained after 8 months of BPA exposure (5–5,000 μg/kg/day) (103). Finally, experimental studies employing T1DM animal models reported increased diabetes incidence following BPA treatment (107–109). In NOD mice, 1 mg/L BPA in drinking water accelerated spontaneous diabetes development, insulitis and islet apoptosis, and decreased numbers of tissue resident macrophages prior to insulitis (107, 108). Similarly to what has been previously described in other studies regarding the BPA non-monotonic dose response, the authors reported that the highest BPA dose (100 mg/L) did produce less severe insulitis than the lowest one (1 mg/L) (107). In parallel, in streptozotocin (STZ)-treated mice, drinking water containing BPA at 1 and 10 mg/L promoted diabetes incidence and affected T-cell immunity (109). Oral gavage administration of BPA 5 mg/kg for 5 days also increased the insulin positive area and transcript expression of estrogen receptors in pancreas, and the hepatic expression of inflammation-related genes in STZ-induced diabetic mice (98).

## A new perspective for the concept of metabolic programming

Over the years steroid hormone research has set the framework for addressing how EDCs may cause endocrine actions. Many biological principles that operate for natural hormones are also central for the mode of action of EDCs. This applies for the activational and organizational concepts. As originally described, organizational effects refer to permanent changes occurring during critical periods of development. They are precisely time-dependent, and affect morphogenesis and differentiation processes of organ systems. It is important to note that some of the organizational effects are not manifested up to later in life, which is in line with the concept of developmental programming. For example, intrauterine exposure to estrogenic compounds is well known to promote abnormalities in the reproductive tract as well as functional changes at puberty and throughout adulthood. This is well exemplified by the case of diethylstilbestrol (DES). Animal studies have shown that perinatal exposure to DES promote a wide range of gene expression changes that have been associated with the increased incidence of neoplasm and vaginal clear cell carcinoma observed in the DES-daughters (110–112). Although initial studies were focused on the reproductive tract, metabolic disturbances may also encompass organizational effects. By contrast, activational effects typically occur in adulthood, are transient in nature, and commonly persist as long as the stimulus is present. An example of this is the effect of abnormal estrogenic activity on adult female fertility (113).

Under this paradigm, we would expect that pregnancy exposure to BPA affect maternal metabolism in a temporary manner whenever the metabolic disruptor were present but this was not the case. We will describe how BPA exposure during pregnancy promotes long-lasting effects in the mothers that were visible months after delivery.

BPA exposed dams (10 or 100 μg/kg/day) from day 9 to 16 of gestation developed glucose intolerance that was more evident in the lowest exposed dose group (70). Moreover, they showed increased insulin resistance, which underlied defects on the insulin signaling cascade. In particular, decreased Akt phosphorylation in response to insulin was found both in liver and skeletal muscle of BPA mums (70, 114). Interestingly, gestational glucose intolerance related to BPA exposure was found to be associated with abnormalities in the tryptophan catabolism. Diet supplementation with vitamin B6 rescued the disrupted metabolic phenotype in the dams (115). It is important to highlight that any degree of glucose intolerance first recognized during pregnancy is diagnosed as GDM. Thus, we can conclude that exposure to low doses of BPA during pregnancy may compromise maternal metabolic status similarly to GDM.

It has also been reported that metabolic disturbances were resolved after parturition but appeared again months later. At 3 months postpartum no differences on glucose tolerance or insulin sensitivity were found between BPA and control dams. However, at 4 months postpartum early symptoms of altered glucose homeostasis showed up. The metabolic status of BPA mice got worse over time and 7 months postpartum animals exhibited enhanced glucose intolerance, severe insulin resistance together with decreased pancreatic β-cell function and mass (114). These findings demonstrate for the first time that a brief exposure to an EDC during pregnancy could have long-term effects on the mother's metabolism and defined a new window of susceptibility for increased incidence of diabetes.

In view of this data, we should keep in mind that metabolic programming induced by EDC exposure could be extended to other critical periods of life as it is the case of pregnancy for the mother. Hence, we should be cautious when considering the importance of the disruption of normal signaling pathways for metabolic memory beyond early development.

## Conclusions

BPA is one of the most pervasive and ubiquitous EDCs. Laboratory animal and human epidemiological studies have revealed the importance of BPA as a contributing factor in the etiology of T2DM. We know that timing of exposure is critical for determining the consequences of exposure to BPA. Developmental period has been outlined as an extremely sensitive window of vulnerability since early-life exposure to this EDC can program the risk for metabolic disorders in adult life. An additional aspect of concern is that the incidence of metabolic abnormalities may increase not only in the exposed individuals but also in the subsequent generations. Some of these multigenerational and transgenerational effects may have an epigenetic origin. Evidence described here also underscore the importance of examining pregnancy, an often understudied critical exposure window for long-term metabolic effects in the mother. Gestational exposure to BPA has been linked to metabolic programming of maternal T2DM, yet more research is needed to understand the molecular mechanisms underlying this phenomenon.

## Author contributions

ET and PA-M wrote the manuscript. RDS, LM, IQ, and EF contributed to the discussion and reviewed and edited the manuscript. All of the authors reviewed and edited the manuscript.

### Conflict of interest statement

The authors declare that the research was conducted in the absence of any commercial or financial relationships that could be construed as a potential conflict of interest.

## References

[1] Ingelfinger JR Jarcho JA. Increase in the incidence of diabetes and its implications. N Engl J Med. (2017) 376:1473–4. 10.1056/NEJMe1616575 28402766

[2] WHO (World Health Organization) (2018). Available online at: http://www.Who.Int/news-room/fact-sheets/detail/diabetes

[3] ADA (American Diabetes Association) Classification and diagnosis of diabetes: Standards of medical care in diabetes-2018. Diab Care (2018) 41:S13–27. 10.2337/dc18-S002 29222373

[4] Kahn SE. The importance of beta-cell failure in the development and progression of type 2 diabetes. J Clin Endocrinol Metab. (2001) 86:4047–58. 10.1210/jcem.86.9.7713 11549624

[5] Chatterjee S Khunti K Davies MJ. Type 2 diabetes. Lancet (2017) 389:2239–51. 10.1016/S0140-6736(17)30058-2 28190580

[6] Zheng Y Ley SH Hu FB. Global aetiology and epidemiology of type 2 diabetes mellitus and its complications. Nat Rev Endocrinol. (2018) 14:88–98. 10.1038/nrendo.2017.151 29219149

[7] Zoeller RT Brown TR Doan LL Gore AC Skakkebaek NE Soto AM. Endocrine-disrupting chemicals and public health protection: A statement of principles from the endocrine society. Endocrinology (2012) 153:4097–110. 10.1210/en.2012-1422 22733974 PMC3423612

[8] Gore AC Chappell VA Fenton SE Flaws JA Nadal A Prins GS. Executive summary to edc-2:the endocrine society's second scientific statement on endocrine-disrupting chemicals. Endocr. Rev. (2015) 36:593–602. 10.1210/er.2015-1093 26414233 PMC4702495

[9] Casals-Casas C Desvergne B. Endocrine disruptors: from endocrine to metabolic disruption. Annu. Rev. Physiol. (2011) 73:135–62. 10.1146/annurev-physiol-012110-142200 21054169

[10] Alonso-Magdalena P Quesada I Nadal A. Endocrine disruptors in the etiology of type 2 diabetes mellitus. Nat. Rev. Endocrinol. (2011) 7:346–53. 10.1038/nrendo.2011.56 21467970

[11] Heindel JJ Blumberg B Cave M Machtinger R Mantovani A Mendez MA. Metabolism disrupting chemicals and metabolic disorders. Reprod Toxicol.(2017) 68:3–33. 10.1016/j.reprotox.2016.10.001 27760374 PMC5365353

[12] Nadal A Quesada I Tuduri E Nogueiras R Alonso-Magdalena P. Endocrine-disrupting chemicals and the regulation of energy balance. Nat Rev Endocrinol. (2017) 13:536–46. 10.1038/nrendo.2017.51 28524168

[13] Dodds EC Lawson W Synthetic estrogenic agents without the phenanthrene nucleus. Nature (1936) 137:996 10.1038/137996a0

[14] Lee I Kim S Kim KT Kim S Park S Lee H. Bisphenol a exposure through receipt handling and its association with insulin resistance among female cashiers. Environ Int. (2018) 117:268–75. 10.1016/j.envint.2018.05.013 29778011

[15] Calafat AM Kuklenyik Z Reidy JA Caudill SP Ekong J Needham LL. Urinary concentrations of bisphenol a and 4-nonylphenol in a human reference population. Environ Health Perspect. (2005) 113:391–5. 10.1289/ehp.7534 15811827 PMC1278476

[16] Vandenberg LN Hauser R Marcus M Olea N Welshons WV. Human exposure to bisphenol a (bpa). Reprod. Toxicol. (2007) 24:139–77. 10.1016/j.reprotox.2007.07.010 17825522

[17] EPA (Environmental Protection Agency) Bisphenol a. Casrn 80-05-7 (2018) Available online at: https://cfpub.Epa.Gov/ncea/iris2/chemicallanding.Cfm?Substance_nmbr=356

[18] vom Saal FS Hughes C An extensive new literature concerning low-dose effects of bisphenol a shows the need for a new risk assessment. Environ Health Perspect. (2005) 113:926–33. 10.1289/ehp.7713 16079060 PMC1280330

[19] EFSA (European Food Safety Authority) (2018). Available online at: https://www.Efsa.Europa.Eu/en/topics/topic/bisphenol

[20] Shelnutt S Kind J Allaben W Bisphenol a: update on newly developed data and how they address ntp's 2008 finding of “some concern”. Food Chem Toxicol. (2013) 57:284–95. 10.1016/j.fct.2013.03.027 23567242

[21] Mimoto MS Nadal A Sargis RM. Polluted pathways: mechanisms of metabolic disruption by endocrine disrupting chemicals. Curr Environ Health Reports (2017) 4:208–22. 10.1007/s40572-017-0137-0 28432637 PMC5921937

[22] Bonefeld-Jorgensen EC Andersen HR Rasmussen TH Vinggaard AM. Effect of highly bioaccumulated polychlorinated biphenyl congeners on estrogen and androgen receptor activity. Toxicology (2001) 158:141–53. 10.1016/S0300-483X(00)00368-1 11275356

[23] Massaad C Barouki R. An assay for the detection of xenoestrogens based on a promoter containing overlapping eres. Environ Health Perspect. (1999) 107:563–6. 10.1289/ehp.99107563 10379002 PMC1566659

[24] Alonso-Magdalena P Ropero AB Soriano S Garcia-Arevalo M Ripoll C Fuentes E. Bisphenol-a acts as a potent estrogen via non-classical estrogen triggered pathways. Mol Cell Endocrinol. (2012) 355:201–7. 10.1016/j.mce.2011.12.012 22227557

[25] Nadal A Fuentes E Ripoll C Villar-Pazos S Castellano-Munoz M Soriano S. Extranuclear-initiated estrogenic actions of endocrine disrupting chemicals: Is there toxicology beyond paracelsus? J Steroid Biochem Mol Biol. (2018) 176:16–22. 10.1016/j.jsbmb.2017.01.014 28159674

[26] Soriano S Ripoll C Alonso-Magdalena P Fuentes E Quesada I Nadal A. Effects of bisphenol a on ion channels: experimental evidence and molecular mechanisms. Steroids (2016) 111:12–20. 10.1016/j.steroids.2016.02.020 26930576

[27] Watson CS Bulayeva NN Wozniak AL Alyea RA. Xenoestrogens are potent activators of nongenomic estrogenic responses. Steroids (2007) 72:124–34. 10.1016/j.steroids.2006.11.002 17174995 PMC1862644

[28] Welshons WV Nagel SC vom Saal FS Large effects from small exposures Iii Endocrine mechanisms mediating effects of bisphenol a at levels of human exposure Endocrinology (2006) 147:S56–69. 10.1210/en.2005-1159 16690810

[29] Wozniak AL Bulayeva NN Watson CS. Xenoestrogens at picomolar to nanomolar concentrations trigger membrane estrogen receptor-alpha-mediated Ca^2+^ fluxes and prolactin release in gh3/b6 pituitary tumor cells. Environ Health Perspect. (2005) 113:431–9. 10.1289/ehp.7505 15811834 PMC1278483

[30] Nadal A Ropero AB Laribi O Maillet M Fuentes E Soria B. Nongenomic actions of estrogens and xenoestrogens by binding at a plasma membrane receptor unrelated to estrogen receptor alpha and estrogen receptor beta. Proc Natl Acad Sci USA. (2000) 97:11603–8. 10.1073/pnas.97.21.11603 11027358 PMC17247

[31] Quesada I Fuentes E Viso-Leon MC Soria B Ripoll C Nadal A. Low doses of the endocrine disruptor bisphenol-a and the native hormone 17beta-estradiol rapidly activate transcription factor creb. FASEB J. (2002) 16:1671–3. 10.1096/fj.02-0313fje 12207000

[32] Alonso-Magdalena P Laribi O Ropero AB Fuentes E Ripoll C Soria B. Low doses of bisphenol a and diethylstilbestrol impair Ca^2+^ signals in pancreatic alpha-cells through a nonclassical membrane estrogen receptor within intact islets of langerhans. Environ Health Perspect. (2005) 113:969–77. 10.1289/ehp.8002 16079065 PMC1280335

[33] Alonso-Magdalena P Morimoto S Ripoll C Fuentes E Nadal A. The estrogenic effect of bisphenol a disrupts pancreatic beta-cell function *in vivo* and induces insulin resistance. Environ Health Perspect. (2006) 114:106–12. 10.1289/ehp.8451 16393666 PMC1332664

[34] Yan S Chen Y Dong M Song W Belcher SM Wang HS. Bisphenol a and 17beta-estradiol promote arrhythmia in the female heart via alteration of calcium handling. PLoS ONE (2011) 6:e25455. 10.1371/journal.pone.0025455 21980463 PMC3181279

[35] Belcher SM Chen Y Yan S Wang HS. Rapid estrogen receptor-mediated mechanisms determine the sexually dimorphic sensitivity of ventricular myocytes to 17beta-estradiol and the environmental endocrine disruptor bisphenol a. Endocrinology (2012) 153:712–20. 10.1210/en.2011-1772 22166976 PMC3275382

[36] Liang Q Gao X Chen Y Hong K Wang HS. Cellular mechanism of the nonmonotonic dose response of bisphenol a in rat cardiac myocytes. Environ Health Perspect. (2014) 122:601–8. 10.1289/ehp.1307491 24569941 PMC4050515

[37] Zsarnovszky A Le HH Wang HS Belcher SM. Ontogeny of rapid estrogen-mediated extracellular signal-regulated kinase signaling in the rat cerebellar cortex: Potent nongenomic agonist and endocrine disrupting activity of the xenoestrogen bisphenol a. Endocrinology (2005) 146:5388–96. 10.1210/en.2005-0565 16123166

[38] Alonso-Magdalena P Ropero AB Carrera MP Cederroth CR Baquie M Gauthier BR. Pancreatic insulin content regulation by the estrogen receptor er alpha. PLoS ONE (2008) 3:e2069. 10.1371/journal.pone.0002069 18446233 PMC2323613

[39] Thomas P Dong J. Binding and activation of the seven-transmembrane estrogen receptor gpr30 by environmental estrogens: a potential novel mechanism of endocrine disruption. J Steroid Biochem Mol Biol. (2006) 102:175–9. 10.1016/j.jsbmb.2006.09.017 17088055

[40] Chevalier N Bouskine A Fenichel P. Bisphenol a promotes testicular seminoma cell proliferation through gper/gpr30. Int J Cancer (2012) 130:241–2. 10.1002/ijc.25972 21312194

[41] Wei J Ding D Wang T Liu Q Lin Y. Mir-338 controls bpa-triggered pancreatic islet insulin secretory dysfunction from compensation to decompensation by targeting pdx-1. FASEB J. (2017) 31:5184–95. 10.1096/fj.201700282R 28774890

[42] Melnick R Lucier G Wolfe M Hall R Stancel G Prins G. Summary of the national toxicology program's report of the endocrine disruptors low-dose peer review. Environ Health Perspect. (2002) 110:427–31. 10.1289/ehp.02110427 11940462 PMC1240807

[43] Vandenberg LN Ehrlich S Belcher SM Ben-Jonathan N Dolinoy DC Eric R Low dose effects of bisphenol A. Endocrine Disrupt. (2013) 1:e26490 10.4161/endo.26490

[44] Vandenberg LN. Non-monotonic dose responses in studies of endocrine disrupting chemicals: bisphenol a as a case study. Dose Response (2014) 12:259–76. 10.2203/dose-response.13-020.Vandenberg 24910584 PMC4036398

[45] Sohoni P Sumpter JP. Several environmental oestrogens are also anti-androgens. J Endocrinol. (1998) 158:327–39. 10.1677/joe.0.1580327 9846162

[46] Moriyama K Tagami T Akamizu T Usui T Saijo M Kanamoto N. Thyroid hormone action is disrupted by bisphenol a as an antagonist. J Clin Endocrinol Metab. (2002) 87:5185–90. 10.1210/jc.2002-020209 12414890

[47] Takayanagi S Tokunaga T Liu X Okada H Matsushima A Shimohigashi Y. Endocrine disruptor bisphenol a strongly binds to human estrogen-related receptor gamma (errgamma) with high constitutive activity. Toxicol Lett. (2006) 167:95–105. 10.1016/j.toxlet.2006.08.012 17049190

[48] Sui Y Ai N Park SH Rios-Pilier J Perkins JT Welsh WJ. Bisphenol a and its analogues activate human pregnane x receptor. Environ Health Perspect. (2012) 120:399–405. 10.1289/ehp.1104426 22214767 PMC3295358

[49] Sargis RM Johnson DN Choudhury RA Brady MJ. Environmental endocrine disruptors promote adipogenesis in the 3t3-l1 cell line through glucocorticoid receptor activation. Obesity (2010) 18:1283–8. 10.1038/oby.2009.419 19927138 PMC3957336

[50] Soriano S Alonso-Magdalena P Garcia-Arevalo M Novials A Muhammed SJ Salehi A. Rapid insulinotropic action of low doses of bisphenol-a on mouse and human islets of langerhans: role of estrogen receptor beta. PLoS ONE (2012) 7:e31109. 10.1371/journal.pone.0031109 22347437 PMC3275611

[51] Hectors TL Vanparys C Pereira-Fernandes A Martens GA Blust R. Evaluation of the ins-1 832/13 cell line as a beta-cell based screening system to assess pollutant effects on beta-cell function. PLoS ONE (2013) 8:e60030. 10.1371/journal.pone.0060030 23555872 PMC3605429

[52] Makaji E Raha S Wade MG Holloway AC. Effect of environmental contaminants on beta cell function. Int J Toxicol. (2011) 30:410–8. 10.1177/1091581811405544 21705745

[53] Lin Y Sun X Qiu L Wei J Huang Q Fang C. Exposure to bisphenol a induces dysfunction of insulin secretion and apoptosis through the damage of mitochondria in rat insulinoma (ins-1) cells. Cell Death Dis. (2013) 4:e460. 10.1038/cddis.2012.206 23328667 PMC3563994

[54] Xin F Jiang L Liu X Geng C Wang W Zhong L. Bisphenol a induces oxidative stress-associated DNA damage in ins-1 cells. Mutat Res Genet Toxicol Environ Mutagen (2014) 769:29–33. 10.1016/j.mrgentox.2014.04.019 25344109

[55] Dai YE Chen W Qi H Liu QQ. Effect of bisphenol a on socs-3 and insulin signaling transduction in 3t3-l1 adipocytes. Mol Med Rep. (2016) 14:331–6. 10.3892/mmr.2016.5224 27176707

[56] Valentino R D'Esposito V Passaretti F Liotti A Cabaro S Longo M. Bisphenol-a impairs insulin action and up-regulates inflammatory pathways in human subcutaneous adipocytes and 3t3-l1 cells. PLoS ONE (2013) 8:e82099. 10.1371/journal.pone.0082099 24349194 PMC3857211

[57] Ariemma F D'Esposito V Liguoro D Oriente F Cabaro S Liotti A. Low-dose bisphenol-a impairs adipogenesis and generates dysfunctional 3t3-l1 adipocytes. PLoS ONE (2016) 11:e0150762. 10.1371/journal.pone.0150762 26942597 PMC4778877

[58] Boucher JG Boudreau A Ahmed S Atlas E *In vitro* effects of bisphenol a beta-d-glucuronide (bpa-g) on adipogenesis in human and murine preadipocytes. Environ. Health Perspect. (2015) 123:1287–93. 10.1289/ehp.1409143 26018136 PMC4671229

[59] Ohlstein JF Strong AL McLachlan JA Gimble JM Burow ME Bunnell BA. Bisphenol a enhances adipogenic differentiation of human adipose stromal/stem cells. J Mol Endocrinol. (2014) 53:345–53. 10.1530/JME-14-0052 25143472 PMC4757902

[60] Grasselli E Cortese K Voci A Vergani L Fabbri R Barmo C. Direct effects of bisphenol a on lipid homeostasis in rat hepatoma cells. Chemosphere (2013) 91:1123–9. 10.1016/j.chemosphere.2013.01.016 23399309

[61] Barker DJ. The fetal and infant origins of disease. Eur J Clin Invest. (1995) 25:457–63. 10.1111/j.1365-2362.1995.tb01730.x 7556362

[62] Barker DJ Martyn CN. The fetal origins of hypertension. Adv Nephrol Necker Hosp. (1997) 26:65–72. 8922125

[63] Hales CN Barker DJ. Type 2 (non-insulin-dependent) diabetes mellitus: the thrifty phenotype hypothesis. Diabetologia (1992) 35:595–601. 10.1007/BF00400248 1644236

[64] Gluckman PD Hanson MA. Living with the past: evolution, development, and patterns of disease. Science (2004) 305:1733–6. 10.1126/science.1095292 15375258

[65] Heindel JJ Vandenberg LN. Developmental origins of health and disease: a paradigm for understanding disease cause and prevention. Curr Opin Pediatr. (2015) 27:248–53. 10.1097/MOP.0000000000000191 25635586 PMC4535724

[66] Alavian-Ghavanini A Ruegg J. Understanding epigenetic effects of endocrine disrupting chemicals: from mechanisms to novel test methods. Basic Clin Pharmacol Toxicol. (2018) 122:38–45. 10.1111/bcpt.12878 28842957

[67] Feil R Fraga MF. Epigenetics and the environment: emerging patterns and implications. Nat Rev Genet. (2012) 13:97–109. 10.1038/nrg3142 22215131

[68] Skinner MK Manikkam M Guerrero-Bosagna C. Epigenetic transgenerational actions of environmental factors in disease etiology. Trends Endocrinol. Metab. (2010) 21:214–22. 10.1016/j.tem.2009.12.007 20074974 PMC2848884

[69] Teperek-Tkacz M Pasque V Gentsch G Ferguson-Smith AC. Epigenetic reprogramming: is deamination key to active DNA demethylation? Reproduction (2011) 142:621–32. 10.1530/REP-11-0148 21911441 PMC3863514

[70] Alonso-Magdalena P Vieira E Soriano S Menes L Burks D Quesada I. Bisphenol a exposure during pregnancy disrupts glucose homeostasis in mothers and adult male offspring. Environ. Health Perspect. (2010) 118:1243–50. 10.1289/ehp.1001993 20488778 PMC2944084

[71] Angle BM Do RP Ponzi D Stahlhut RW Drury BE Nagel SC Metabolic disruption in male mice due to fetal exposure to low but not high doses of bisphenol a (bpa): evidence for effects on body weight, food intake, adipocytes, leptin, adiponectin, insulin and glucose regulation. Reproduct Toxicol. (2013) 42:256–68. 10.1016/j.reprotox.2013.07.017 PMC3886819 23892310

[72] Liu J Yu P Qian W Li Y Zhao J Huan F. Perinatal bisphenol a exposure and adult glucose homeostasis: identifying critical windows of exposure. PLoS ONE (2013) 8:e64143. 10.1371/journal.pone.0064143 23675523 PMC3651242

[73] Fang F Gao Y Wang T Chen D Liu J Qian W. Insulin signaling disruption in male mice due to perinatal bisphenol a exposure: role of insulin signaling in the brain. Toxicol Lett. (2016) 245:59–67. 10.1016/j.toxlet.2016.01.007 26779933

[74] Mackay H Patterson ZR Khazall R Patel S Tsirlin D Abizaid A. Organizational effects of perinatal exposure to bisphenol-a and diethylstilbestrol on arcuate nucleus circuitry controlling food intake and energy expenditure in male and female cd-1 mice. Endocrinology (2013) 154:1465–75. 10.1210/en.2012-2044 23493373

[75] MacKay H Patterson ZR Abizaid A. Perinatal exposure to low-dose bisphenol-a disrupts the structural and functional development of the hypothalamic feeding circuitry. Endocrinology (2017) 158:768–77. 10.1210/en.2016-1718 28323920

[76] Song S Zhang L Zhang H Wei W Jia L. Perinatal bpa exposure induces hyperglycemia, oxidative stress and decreased adiponectin production in later life of male rat offspring. Int J Environ Res Public Health (2014) 11:3728–42. 10.3390/ijerph110403728 24705360 PMC4025022

[77] Veiga-Lopez A Moeller J Sreedharan R Singer K Lumeng C Ye W. Developmental programming: Interaction between prenatal bpa exposure and postnatal adiposity on metabolic variables in female sheep. Am J Physiol Endocrinol Metab. (2016) 310:E238–47. 10.1152/ajpendo.00425.2015 26646100 PMC4888526

[78] Ryan KK Haller AM Sorrell JE Woods SC Jandacek RJ Seeley RJ. Perinatal exposure to bisphenol-a and the development of metabolic syndrome in cd-1 mice. Endocrinology (2010) 151:2603–12. 10.1210/en.2009-1218 20351315 PMC2875828

[79] van Esterik JC Dolle ME Lamoree MH van Leeuwen SP Hamers T Legler J Programming of metabolic effects in c57bl/6jxfvb mice by exposure to bisphenol a during gestation and lactation. Toxicology (2014) 321:40–52. 10.1016/j.tox.2014.04.001 24726836

[80] van Esterik JC Vitins AP Hodemaekers HM Kamstra JH Legler J Pennings JL Liver DNA methylation analysis in adult female c57bl/6jxfvb mice following perinatal exposure to bisphenol a. Toxicol Lett. (2015) 232:293–300. 10.1016/j.toxlet.2014.10.021 25455458

[81] Garcia-Arevalo M Alonso-Magdalena P Servitja JM Boronat-Belda T Merino B Villar-Pazos S. Maternal exposure to bisphenol-a during pregnancy increases pancreatic beta-cell growth during early life in male mice offspring. Endocrinology (2016) 157:4158–71. 10.1210/en.2016-1390 27623287

[82] Whitehead R Guan H Arany E Cernea M Yang K. Prenatal exposure to bisphenol a alters mouse fetal pancreatic morphology and islet composition. Horm Mol Biol Clin Investig. (2016) 25:171–9. 10.1515/hmbci-2015-0052 26812801

[83] Wei J Lin Y Li Y Ying C Chen J Song L. Perinatal exposure to bisphenol a at reference dose predisposes offspring to metabolic syndrome in adult rats on a high-fat diet. Endocrinology (2011) 152:3049–61. 10.1210/en.2011-0045 21586551

[84] Garcia-Arevalo M Alonso-Magdalena P Rebelo Dos Santos J Quesada I Carneiro EM Nadal A. Exposure to bisphenol-a during pregnancy partially mimics the effects of a high-fat diet altering glucose homeostasis and gene expression in adult male mice. PLoS ONE (2014) 9:e100214. 10.1371/journal.pone.0100214 24959901 PMC4069068

[85] Cabaton NJ Canlet C Wadia PR Tremblay-Franco M Gautier R Molina J. Effects of low doses of bisphenol a on the metabolome of perinatally exposed cd-1 mice. Environ Health Perspect. (2013) 121:586–93. 10.1289/ehp.1205588 23425943 PMC3673190

[86] Tremblay-Franco M Cabaton NJ Canlet C Gautier R Schaeberle CM Jourdan F. Dynamic metabolic disruption in rats perinatally exposed to low doses of bisphenol-a. PLoS ONE (2015) 10:e0141698. 10.1371/journal.pone.0141698 26517871 PMC4627775

[87] Ma Y Xia W Wang DQ Wan YJ Xu B Chen X. Hepatic DNA methylation modifications in early development of rats resulting from perinatal bpa exposure contribute to insulin resistance in adulthood. Diabetologia (2013) 56:2059–67. 10.1007/s00125-013-2944-7 23748860

[88] Li G Chang H Xia W Mao Z Li Y Xu S. F0 maternal bpa exposure induced glucose intolerance of f2 generation through DNA methylation change in gck. Toxicol Lett. (2014) 228:192–9. 10.1016/j.toxlet.2014.04.012 24793715

[89] Susiarjo M Xin F Bansal A Stefaniak M Li C Simmons RA. Bisphenol a exposure disrupts metabolic health across multiple generations in the mouse. Endocrinology (2015) 156:2049–58. 10.1210/en.2014-2027 25807043 PMC4430620

[90] Mao Z Xia W Chang H Huo W Li Y Xu S. Paternal bpa exposure in early life alters igf2 epigenetic status in sperm and induces pancreatic impairment in rat offspring. Toxicol Lett. (2015) 238:30–8. 10.1016/j.toxlet.2015.08.009 26276081

[91] Bansal A Rashid C Xin F Li C Polyak E Duemler A. Sex- and dose-specific effects of maternal bisphenol a exposure on pancreatic islets of first- and second-generation adult mice offspring. Environ Health Perspect. (2017) 125:097022. 10.1289/EHP1674 29161229 PMC5915189

[92] Manikkam M Guerrero-Bosagna C Tracey R Haque MM Skinner MK. Transgenerational actions of environmental compounds on reproductive disease and identification of epigenetic biomarkers of ancestral exposures. PLoS ONE (2012) 7:e31901. 10.1371/journal.pone.0031901 22389676 PMC3289630

[93] Manikkam M Tracey R Guerrero-Bosagna C Skinner MK. Plastics derived endocrine disruptors (bpa, dehp and dbp) induce epigenetic transgenerational inheritance of obesity, reproductive disease and sperm epimutations. PLoS ONE (2013) 8:e55387. 10.1371/journal.pone.0055387 23359474 PMC3554682

[94] Bodin J Bolling AK Becher R Kuper F Lovik M Nygaard UC. Transmaternal bisphenol a exposure accelerates diabetes type 1 development in nod mice. Toxicol Sci. (2014) 137:311–23. 10.1093/toxsci/kft242 24189131

[95] Malaise Y Menard S Cartier C Gaultier E Lasserre F Lencina C. Gut dysbiosis and impairment of immune system homeostasis in perinatally-exposed mice to bisphenol a precede obese phenotype development. Sci Rep. (2017) 7:14472. 10.1038/s41598-017-15196-w 29101397 PMC5670173

[96] Batista TM Alonso-Magdalena P Vieira E Amaral ME Cederroth CR Nef S. Short-term treatment with bisphenol-a leads to metabolic abnormalities in adult male mice. PLoS ONE (2012) 7:e33814. 10.1371/journal.pone.0033814 22470480 PMC3314682

[97] Jayashree S Indumathi D Akilavalli N Sathish S Selvaraj J Balasubramanian K. Effect of bisphenol-a on insulin signal transduction and glucose oxidation in liver of adult male albino rat. Environ Toxicol Pharmacol. (2013) 35:300–10. 10.1016/j.etap.2012.12.016 23376180

[98] Kang HS Yang H Ahn C Kang HY Hong EJ Jaung EB. Effects of xenoestrogens on streptozotocin-induced diabetic mice. J Physiol Pharmacol. (2014) 65:273–82. 24781736

[99] Marmugi A Ducheix S Lasserre F Polizzi A Paris A Priymenko N. Low doses of bisphenol a induce gene expression related to lipid synthesis and trigger triglyceride accumulation in adult mouse liver. Hepatology (2012) 55:395–407. 10.1002/hep.24685 21932408

[100] Corkey BE Banting lecture 2011: Hyperinsulinemia: cause or consequence? Diabetes (2012) 61:4–13. 10.2337/db11-1483 22187369 PMC3237642

[101] Ahangarpour A Afshari G Mard SA Khodadadi A Hashemitabar M. Preventive effects of procyanidin a2 on glucose homeostasis, pancreatic and duodenal homebox 1, and glucose transporter 2 gene expression disturbance induced by bisphenol a in male mice. J Physiol Pharmacol. (2016) 67:243–52. 27226184

[102] Veissi M Jafarirad S Ahangarpour A Mohaghegh SM Malehi AS. Co-exposure to endocrine disruptors: effect of bisphenol a and soy extract on glucose homeostasis and related metabolic disorders in male mice. Endocr Regul. (2018) 52:76–84. 10.2478/enr-2018-0009 29715189

[103] Marmugi A Lasserre F Beuzelin D Ducheix S Huc L Polizzi A. Adverse effects of long-term exposure to bisphenol a during adulthood leading to hyperglycaemia and hypercholesterolemia in mice. Toxicology (2014) 325:133–43. 10.1016/j.tox.2014.08.006 25168180

[104] Moon MK Jeong IK Jung Oh T Ahn HY Kim HH Park YJ. Long-term oral exposure to bisphenol a induces glucose intolerance and insulin resistance. J Endocrinol. (2015) 226:35–42. 10.1530/JOE-14-0714 25972359

[105] Perreault L McCurdy C Kerege AA Houck J Faerch K Bergman BC. Bisphenol a impairs hepatic glucose sensing in c57bl/6 male mice. PLoS ONE (2013) 8:e69991. 10.1371/journal.pone.0069991 23922885 PMC3726717

[106] Moghaddam HS Samarghandian S Farkhondeh T. Effect of bisphenol a on blood glucose, lipid profile and oxidative stress indices in adult male mice. Toxicol Mech Methods (2015) 25:507–13. 10.3109/15376516.2015.1056395 26376105

[107] Bodin J Bolling AK Samuelsen M Becher R Lovik M Nygaard UC. Long-term bisphenol a exposure accelerates insulitis development in diabetes-prone nod mice. Immunopharmacol Immunotoxicol. (2013) 35:349–58. 10.3109/08923973.2013.772195 23496298

[108] Bodin J Kocbach Bolling A Wendt A Eliasson L Becher R Kuper F Exposure to bisphenol a, but not phthalates, increases spontaneous diabetes type 1 development in nod mice. Toxicol Reports (2015) 2:99–110. 10.1016/j.toxrep.2015.02.010 PMC5598488 28962342

[109] Cetkovic-Cvrlje M Thinamany S Bruner KA. Bisphenol a (bpa) aggravates multiple low-dose streptozotocin-induced type 1 diabetes in c57bl/6 mice. J Immunotoxicol. (2017) 14:160–8. 10.1080/1547691X.2017.1334722 28707492

[110] Newbold R. Cellular and molecular effects of developmental exposure to diethylstilbestrol: implications for other environmental estrogens. Environ Health Perspect. (1995) 103 (Suppl 7):83–7. 8593881 10.1289/ehp.95103s783 PMC1518878

[111] Newbold RR Jefferson WN Grissom SF Padilla-Banks E Snyder RJ Lobenhofer EK. Developmental exposure to diethylstilbestrol alters uterine gene expression that may be associated with uterine neoplasia later in life. Mol Carcinog. (2007) 46:783–96. 10.1002/mc.20308 17394237 PMC2254327

[112] Reed CE Fenton SE. Exposure to diethylstilbestrol during sensitive life stages: a legacy of heritable health effects. Birth Defects Res C Embryo Tod. (2013) 99:134–46. 10.1002/bdrc.21035 23897597 PMC3817964

[113] Vandenberg LN Colborn T Hayes TB Heindel JJ Jacobs DRJr Lee DH. Hormones and endocrine-disrupting chemicals: Low-dose effects and nonmonotonic dose responses. Endocr Rev. (2012) 33:378–455. 10.1210/er.2011-1050 22419778 PMC3365860

[114] Alonso-Magdalena P Garcia-Arevalo M Quesada I Nadal A. Bisphenol-a treatment during pregnancy in mice: a new window of susceptibility for the development of diabetes in mothers later in life. Endocrinology (2015) 156:1659–70. 10.1210/en.2014-1952 25830705

[115] Susiarjo M Xin F Stefaniak M Mesaros C Simmons RA Bartolomei MS. Bile acids and tryptophan metabolism are novel pathways involved in metabolic abnormalities in bpa-exposed pregnant mice and male offspring. Endocrinology (2017) 158:2533–42. 10.1210/en.2017-00046 28549143 PMC5551548

