# Supplementary material for: Timing of Exposure and Bisphenol-A: Implications for Diabetes Development
Source: Front Endocrinol (Lausanne). 2018 Oct 31;9:648. doi: 10.3389/fendo.2018.00648 (PMC6220716; doi:10.3389/fendo.2018.00648)
Supplement: Supplementary file 1 [file Table_1.docx]

**Supplemental table 1. Summary of the *in vivo* studies examining the association between BPA and diabetes at different timing of exposure**

| **Specie and gender** | **Dose** | **Exposure time** | **Route of administration/ vehicle** | **Age at the moment of the study** | **Main effects** | **Refs** |
| --- | --- | --- | --- | --- | --- | --- |
| NMRI male mice | 100 μg/kg/d | 20 days | S.c. inject/ethyl alcohol 0.1% | 2.5-3 months old | Increased fasting blood glucose, decreased plasma insulin levels, reduced pancreatic Pdx1 and Glut 2 expression | Ahangarpour A et al, Journal of physiology and pharmacology 2016 |
| OF1 male mice | 50 μg/kg/d | Two injections daily during 4 days | S.c. inject/corn oil | 2-2.5 months old | Glucose intolerance, insulin resistance, hyperinsulinemia | Alonso-Magdalena et al, Environmental health perspectives 2006 |
| Pregnant OF-1 mice | 10 or 100 μg/kg/d | GD9-GD16 | S.c. inject/corn oil | GD16-GD18 (mums) | Hyperinsulinemia, increased plasma triglyceride, glycerol and leptin levels | Alonso-Magdalena et al, Environmental health perspectives 2010 |
|  |  |  |  | 4 months after delivery (mums) | Increased body weight and plasma insulin, triglycerides, glycerol and leptin levels. Impaired glucose tolerance and reduced insulin sensitivity |  |
|  |  |  |  | 6 months (male offspring) | Impaired glucose tolerance and insulin resistance |  |
| Pregnant OF-1 mice | 10 or 100 μg/kg/d | GD9-GD16 | S.c. inject/corn oil | 6 and 7 months after delivery (mums) | Increased body weight, impaired glucose tolerance, insulin resistance, decreased pancreatic β-cell mass and insulin secretion | Alonso-Magdalena et al, Endocrinology 2015 |
| Pregnant CD-1 mice | 5, 50, 500, 5,000 or 50,000 μg/kg/d | GD9-GD18 | Orally fed with micropipetter/corn oil | 18-19 weeks old (offspring) | Age-related change in food intake, increased body weight, liver weight, abdominal adipocyte mass, adipocyte number and volume, and serum leptin and insulin levels. Reduced adiponectin levels. Impaired glucose tolerance | Angle et al, Reproductive toxicology 2013 |
| Pregnant C57BL/6 | 10 μg/kg/d or 100 mg/kg/d | From 2 weeks prior to mating until weaning | Diet | 16-21 weeks old (male F1 and F2 offspring) | Impaired insulin secretion, reduced β-cell mass, increased β-cell death, levels of pro-inflammatory cytokines and islet Igf2 expression. Altered islet DNA methylation | Bansal et al, Environmental health perspectives 2017 |
| OF1 male mice | 100 μg/kg/d | 8 days | S.c. inject/corn oil | 3 months old | Increased insulin secretion, impaired insulin sensitivity, decreased food intake, body temperature and locomotor activity. Reduced Akt phosphorylation and impaired MAPK signaling in skeletal muscle. Up-regulated IRS-1 protein levels in skeletal muscle and liver | Batista et al, Plos One 2012 |
| NOD female mice | 1 or 100 mg/L | From 4 to 12 weeks old | Drinking water | 7-12 weeks old | Higher glycemia levels, increased insulitis and apoptotic cells, decreased number of tissue resident macrophages in pancreatic islets | Bodin et al, Immunopharmacology and immunotoxicology 2013 |
| Pregnant NOD mice | 0.1, 1 or 10 mg/L | GD0-PND21 | Drinking water | 7-28 weeks old female offspring | Increased α and β-cells and tissue resident macrophage apoptosis, augmented number of regulatory T cells and active caspase-3 positive cells in pancreatic islets. Increased severity of insulitis and incidence of diabetes | Bodin et al, Toxicological sciences 2014 |
| Pregnant CD1 mice | 0.025, 0.25 or 25 μg/kg/d | GD8- Day 16 of lactation | Subcutaneous osmotic pump/DMSO | PND2, PND21 | Changes in the metabolome: variations in glucose, pyruvate, some amino acids, and neurotransmitters (γ-aminobutyric acid and glutamate) | Cabaton et al, Environmental health perspectives 2013 |
| C57BL6 male mice | 1 or 10 mg/L | From 4 to 16 weeks old | Drinking water | 10-17 weeks old | Decreased splenic T-cell subpopulation and levels of INFγ and TNFα. Elevation of splenic T-cell formation of proinflammatory cytokines. Aggravation of T1D development | Cetkovic-Cvrlej et al, Journal of immunotoxicology 2017 |
| Pregnant C57BL6 mice | 100 μg/kg/d | GD6-PND0 | S.c. inject/corn oil | 8 months old (male offspring) | Impaired insulin signaling in the brain: decreased mRNA expression of Glut (1/3/4), diminished expression of phosphorylated IR, AKT, GDK3b and ERK proteins | Fang et al, Toxicology letters 2016 |
|  |  | PND0-PND21 |  |  |  |  |
|  |  | GD6-PND21 |  |  |  |  |
| Pregnant OF-1 mice | 10 μg/kg/d | GD9-GD16 | S.c. inject/corn oil | 17 weeks old (male offspring) | Chow diet: increased fasting blood glucose, insulin secretion and NEFA levels. In WAT, decreased mRNA expression of Srebp1c, Pparα and Cpt1β. In liver increased mRNA expression of Pparγ and Prkaa1, triglyceride content and decreased Cd36. HFD: reduced retroperitoneal and perigonadal fat pad weight, decreased insulin secretion and Cpt1α mRNA expression in skeletal muscle. | Garcia-Arevalo et al, Plos One 2014 |
|  |  |  |  | 28 weeks old (male offspring) | Chow diet: increased body weight, fasting blood glucose, perigonadal fat pad weight and NEFA. Glucose intolerance. HFD: reduced insulin secretion, increased food intake |  |
| Pregnant OF-1 mice | 10 or 100 μg/kg/d | GD9-GD16 | S.c. inject/corn oil | PND0, PND21, PND30 and PND120 (male offspring) | PND0 and PND21: increased β-cell mass PND30: hyperinsulimenia, increased β-cell mass, expression of genes involved in cell cycle, pancreatic β-cell proliferation, plasma leptin and decreased β-cell apoptosis. PND120: decreased β-cell mass and fasting glycemia levels | Garcia-Arevalo et al, Endocrinology 2016 |
| Wistar albino male rats | 20 or 200 mg/kg/d | 30 days | Orally/corn oil | Adulthood | Increased insulin levels. Decreased testosterone levels, hepatic glucose oxidation and glycogen content | Jayashree et al, Environmental toxicology and pharmacology 2013 |
| Male ICR mice | 5 mg/kg/d | 5 days | Gavage/corn oil | 10 weeks old | Increased plasma insulin levels and pancreatic β-cell area | Kang et al, Journal of physiology and pharmacology 2014 |
| Pregnant SD rats | 40 μg/kg/d | GD0-PND21 | Orally/corn oil | 16 weeks old (F1 offspring) | Decreased global methylation in sperm | Li et al, Toxicology letters 2014 |
|  |  |  |  | 12, 23 and 25 weeks old (F2 offspring) | Glucose intolerance, insulin resistance, increased fasting insulin levels, downregulation of Gck gene, DNA methylation change in hepatic Gck |  |
| Pregnant C57BL6 mice | 100 μg/kg/d | GD1-GD6 | S.c. inject/corn oil | 3, 6 and 8 months old (offspring) | Changes on body weight, glucose intolerance, decreased insulin sensitivity and impairment of pancreatic β-cell function depending on the dose and the exposure timing | Liu et al, Plos One 2013 |
|  |  | GD6-PND0 |  |  |  |  |
|  |  | PND0-PND21 |  |  |  |  |
|  |  | GD6-PND21 |  |  |  |  |
| Pregnant Wistar rats | 50 μg/kg/d | GD0-PND21 | Oral gavage/corn oil | 3 and 21 weeks old (offspring) | Increased insulin levels, impaired insulin sensitivity, decreased hepatic global DNA methylation, Gck gene expression and glycogen storage | Ma et al, Diabetologia 2013 |
| Pregnant CD1 mice | 0.19, 0.36, 3.49 or 7.2 μg/kg/d | GD0-PND21 | Diet | 3, 6 and 8.5 months old (offspring) | Impaired glucose tolerance, dimorphic alterations in the structure of hypothalamic energy balance circuitry | Mackay et al, Endocrinology 2013 |
| Pregnant CD1 mice | 20 μg/kg/d | GD0-PND21 | Diet | Different PN days (PND2, 8, 10,12,16 and 21) | Reduced density of POMC projections into PVN and delayed postnatal surges | Mackay et al, Endocrinology 2017 |
| Pregnant C3H/HeN mice | 50 μg/kg/d | GD15-PND21 | Orally/0.1% ethanol in corn oil | From weaning to PND170 | Impaired glucose tolerance, decreased insulin sensitivity, gonadal WAT inflammation, local and systemic immune homeostasis disturbances | Malaise et al, Scientific Reports 2017 |
| Pregnant rats | 40 μg/kg/d | GD0-PND21 | Orally/corn oil | Birth, 3 and 21 weeks old (F2 offspring) and 8 weeks old (F1 offspring) | F2 offspring: Glucose intolerance, decreased pancreatic β-cell mass, expression of Igf2 and associated hypermethylation in islets. F1 offspring: abnormal expression and methylation of Igf2 in sperm | Mao et al, Toxicology letters 2015 |
| CD1 male mice | 5, 50, 500 or 5,000 μg/kg/d | 28 days | Diet | 10 weeks old | Increased plasma insulin levels, hepatic mRNA and protein related to lipid biosynthesis and lipid content | Marmugi et al, Hepatology 2012 |
| CD1 male mice | 5, 50, 500 or 5,000 μg/kg/d | 8 months | Drinking water | 3, 5.6 and 9.5 months old | Altered glucose tolerance. Increased perigonadal WAT, glucose, insulin, cholesterol and LDL levels and hepatic gene expression related to cholesterol biosynthesis | Marmugi et al, Toxicology 2014 |
| Male mice | 0.5 or 2 mg/kg | 4 weeks | Intraperitoneally inject/olive oil | Adulthood | Increased glycemia, cholesterol, tryglicerides, LDL, HDL and MDA levels. Decreased levels of GSH and TAS | Moghaddam et al, Toxicology mechanisms and methods 2015 |
| C57BL6 male mice | 50 μg/kg/d | 12 weeks | Drinking water | 18 weeks old | Glucose intolerance, decreased Akt phosphorylation in skeletal muscle | Moon et al, The Journal of endocrinology 2015 |
| C57BL6 male mice | 50 μg/kg/d | 2 weeks | Drinking water | 6 and 6.5 months old | Diminished glucokinase activity after acute exposure (two hours after BPA administration) and chronic exposure (two weeks) | Perreault et al, Plos One 2013 |
| Pregnant CD1 mice | 0.25 μg/kg/d | GD0-PND21 | Diet | Several time points from 3 to 15 weeks old | Pups heavier at the moment of weaning and longer at 4 weeks old. No changes on glucose tolerance | Ryan et al, Endocrinology 2010 |
| Pregnant Sprague-Dawley rat | 10 μg/mL | GD6-PND21 | Drinking water | PND1-PND100 (male offspring) | Insulin resistance, increased glucose levels, oxidative stress and decreased adiponectin levels in puberty and adult stage | Song et al, International journal of environmental research and public health 2014 |
| Pregnant C57BL/6 mice | 10 μg/kg/d or 10 mg/kg/d | 2 weeks before mating-PND21 | Diet | Mums GD16.5-17.5 and F1 and F2 male offspring at different time points from E16.5 to PND117 | Mums: glucose intolerance | Susiarjo et al, Endocrinology 2015 |
|  |  |  |  |  | F1 male offspring: PND21 reduced body weight, accelerated body weight postweaning. PND97-PND117 increased body weight, body fat content and insulin levels, and glucose intolerance |  |
|  |  |  |  |  | F2 male offspring: Higher body fat content, glucose intolerance, reduced glucose-stimulated insulin secretion, increased Igf2 mRNA expression and DNA methylation at CpG site 2 of the DMR1 in embryos |  |
| Pregnant C57BL/6 mice | 10 μg/kg/d or 10 mg/kg/d | 2 weeks before mating-GD18.5 | Diet | Mums GD16.5 Offspring E18.5 | Mums: increased tryptophan levels, improvement of glucose tolerance after vit B6 supplementation | Susiarjo et al, Endocrinology 2017 |
|  |  |  |  |  | Offspring: Dose-sensitive changes in hepatic metabolome, altered bile acid metabolism, increased tryptophan levels |  |
| Pregnant SD rats | 0.25, 2.5, 25 or 250 μg/kg/d | GD9-PND16 | Subcutaneous osmotic pump/DMSO | PND21, PND50, PND90, PND140 and PND200 | Time/age dependent metabolome changes | Tremblay-Franco et al, Plos One 2015 |
| Pregnant C57BL/6JxFVB | 0-3,000 μg/kg/d | 2 weeks before mating-PND21 | Diet | 17 and 23 weeks of age (offspring) | Male offspring: dose-dependent increment of body and liver weights and decrease in glucagon levels | van Esterik et al, Toxicology 2014 |
|  |  |  |  |  | Female offspring:dose-dependent increment of body weight, liver, muscle and fat pad weights, adipocyte size, serum leptin,adiponectin and lipids |  |
| Pregnant C57BL/6JxFVB | 0-3,000 μg/kg/d | 2 weeks before mating-PND21 | Diet | 23 weeks of age (offspring) | No effect of global hepatic DNA methylation | van Esterik et al, Toxicoloy letters 2015 |
| Pregnant sheep | 50, 500 or 5,000 μg/kg/d | GD30-GD90 | S.c. inject/corn oil | Different time points from 6 weeks to 15 months of age (female offspring) | Prepubertal: higher fasting glucose levels | Veiga-Lopez et al, American journal of physiology Endocrinology and metabolism 2016 |
|  |  |  |  |  | Postpubertal: decreased insulin sensitivity, higher rate of visceral vs subcutaneous fat, larger area and diameter of visceral adipocytes, reduced mRNA adiponectin expression, increased CD68 in subcutaneous adipose tissue |  |
| NMRI male mice | 100 μg/kg/d | 4 weeks | S.c. inject/0.1 % ethanol in distilled water | 13-13.5 weeks of age | Increased fasting blood glucose levels, decreased insulin and adiponectin levels and HOMA-b | Veissi et al, Endocrine regulations 2018 |
| Pregnant Wistar rats | 50, 250 or 1,250 μg/kg/d | GD0-PND21 | Oral gavage/corn oil | Different time points from birth to 26 weeks of age (offspring) | Chow Diet: increased body weight and insulin levels. Glucose intolerance. HFD: increased body weight and glucose, insulin and adiponectin levels. Glucose intolerance and dyslipidemia | Wei et al, Endocrinology 2011 |
| C57BL/6 male mice | 50 or 500 μg/kg/d | 8 weeks | Oral gavage/corn oil | 11 weeks of age | Impaired glucose tolerance, increased insulin secretion and Pdx1 expression | Wei et al, FASEB 2017 |
| Pregnant C57BL/6 mice | 25 mg BPA/kg diet | E7.5-E8.5 | Diet | E18.5 | Increased number of islet-cell clusters, glucagon expression in islets and the number of glucagon-expressing islet cell-clusters | Whitehead et al, Hormone molecular biology and clinical investigation 2016 |
